# Adherence to standardized assessments through a complexity-based model for categorizing rehabilitation©: design and implementation in an acute hospital

**DOI:** 10.1186/s12911-018-0590-1

**Published:** 2018-03-12

**Authors:** Tania Gutiérrez Panchana, Viviane Hidalgo Cabalín

**Affiliations:** Physical Medicine and Rehabilitation, Clínica Alemana , Santiago, Chile

**Keywords:** Categorization, Standardized assessment, Clinical adherence, Physical therapists

## Abstract

**Background:**

The use of measurement instruments has become a major issue in physical therapy, but their use in daily practice is rare. The aim of this paper is to describe adherence to standardized assessments by physical therapists using a complexity-based model for categorizing rehabilitation (CMCR) at the Clínica Alemana of Santiago, an acute hospital in Chile.

**Methods:**

This retrospective cohort study used 145,968 participant records that were stored in the inpatient database between July 2011 and December 2015. Adherence to the CMCR by 31 physical therapists working with intensive care unit (ICU) and non-ICU inpatients was assessed every quarter using the electronic patient records (EPR). This instrument (CMCR) linked clinical functional assessment to the degree of severity, thereby setting a score used to categorize patients as low, medium and high complexity. 96,400 instances of inpatient care where the physician recommended physical therapy were categorized. This was from a total of 145,968 instances of inpatient care recorded throughout the duration of the study (17 quarters). Trends in adherence were analyzed using a Prais-Winsten regression (a first-order autoregressive model). The trends were compared using a repeated measures ANOVA for mixed models with a significance level of 0.05. The use of the CMCR was included as one of the organization’s quality indicators associated with the hospital’s accreditation processes.

**Results:**

Adherence increased by 1.48% every quarter (*p* = 0.005) for both ICU and non-ICU patients. On average, adherence with ICU patients was 16.98% greater than with non-ICU patients. Although adherence was always greater with ICU patients, the rate of increase with non-ICU patients was significantly greater: 1.62% (*p* = 0.007) vs. 1.28% (*p* = 0.003), respectively.

**Conclusion:**

The CMCR facilitated adherence to standardized assessments by physical therapists working with ICU and non-ICU inpatients in an acute hospital, while linking this instrument to the organization’s quality management process proved to be an effective strategy for the duration of this study (17 quarters).

**Electronic supplementary material:**

The online version of this article (10.1186/s12911-018-0590-1) contains supplementary material, which is available to authorized users.

## Background

The use of assessment tools in clinical practice is important for both clinical rehabilitation professionals and patients. It is also essential when it comes to showing results [1]. The use of assessment scales contributes to improving clinical reasoning, objectifying and quantifying the interventions, which is essential for a good clinical practice [2–4].

Despite these benefits, the standardized use of clinical assessment scales in rehabilitation remains a challenge. In their study, Jette et al. interviewed 1000 physical therapists, all members of the American Physical Therapy Association (APTA). That study revealed that only 48% of physical therapists used assessment scales during their daily clinical practice, as they felt it improved communication with patients as well as care planning. The remaining 52% indicated that they did not use assessment scales with their patients [5]. A number of papers have described the barriers preventing rehabilitation professionals from using routine assessment tools. These include lack of time allocated for the application, lack of knowledge of the available scales, lack of clarity to interpret the results, and even problems inherent to the healthcare provider [6–9]. The literature notes the need for rehabilitation professionals to assemble a core set of assessment instruments, which can be easily applied, and where the results obtained can be clearly interpreted. To the best of our knowledge, there is no clinical model in Chile that allows the application of assessment scales in physical therapy to be standardized. The literature has shown that continuous training and systematic feedback on the benefits of using assessment scales improves adherence among rehabilitation professionals [10].

As a consequence of the national and international accreditation processes in the Clínica Alemana’s Rehabilitation Service, a lack of records was detected, which led to the need to implement an innovation strategy to increase the use of standardized and systematic assessments with inpatients. In view of this need, the complexity-based model for categorizing rehabilitation (CMCR) was designed. It was meant to facilitate the use of standardized clinical assessments by physical therapists.

The objective of this research was to describe the adherence of physical therapists to the use of standardized assessment by means of a CMCR at an acute hospital.

## Method

This retrospective cohort study used 145,968 participant records that were stored in the inpatient database between July 2011 and December 2015. All of the participants were inpatients at the Clínica Alemana of Santiago. They were prescribed physical therapy by the physician and then categorized by the physical therapist, using standardized assessments via the CMCR.

The CMCR was developed using a matrix of clinical variables. To design the matrix, the rehabilitation teams were grouped by medical specialty (Neurorehabilitation, Adult ICU, Pulmonary Rehabilitation, Pediatric Rehabilitation, Motor Rehabilitation, Neonatology, Orthopedic Surgery, Geriatrics, Oncology and Cardiac Rehabilitation). Subsequently, based on their clinical experience and available evidence, the teams were then asked to select and share standardized clinical assessments to be used with an inpatient.

The matrix has two axes: the vertical axis provides the functional clinical assessment to be applied during the patient’s rehabilitation, while the horizontal axis shows its 3 severity levels. The lowest severity level is column X, where each cell has a score of 1 point. The sum of X (Σx) provides the total number of points with respect to the variables chosen. Table 1 shows 4 clinical variables chosen, so Σx is 4. Then, the next severity level is column Y, where each cell has a score of 2 points: as there are 4 variables, the sum of Y (Σy) is 8. Finally, the highest severity level is column Z, where each cell has a score of 3 points, so the sum of Z (Σz) is 12 (Table 1). The lowest complexity is obtained from the sum of column X, Total Score (TS) = Σx, medium complexity is obtained from the range of Σx + 1 ≤ TS ≤ Σy, and the highest complexity, from the range obtained of Σy + 1 ≤ TS ≤ Σz (Table 1). Each complexity is associated with a therapeutic load (number of sessions) in order to take the patient from the highest to the lowest complexity level within the shortest possible time. In the interests of the aforementioned categorization, the professionals were asked to fill out the instrument within 15–30 min, depending on the scales available in each matrix.

**Table 1:** Categorization Matrix

LEVEL OF SEVERITY				
Clinical Variables	Variables	X (1 point)	Y (2 points)	Z (3 points)
	Clinical Variable A			
	Clinical Variable B			
	Clinical Variable C			
	Clinical Variable D			
	Total Score	ΣX	ΣY	ΣZ
CATEGORIZATION				
	Low Complexity		TS^a^ = ΣX	
	Medium Complexity		Σx+1≤TS ≤Σy	
	High Complexity		Σy+1≤TS ≤Σz	

Rows: Functional clinical variables. Columns: Level of severity ^(a)^TS: total score

The management of the same complexities will vary according to the clinical variable that determines the issues in the patient; therefore, we can have patients of the same complexity being attended with different protocols. For example, in the patients categorized with the ICU mobility matrix, the medium complexity could be a result of the change the state of consciousness, which is why the protocols to apply will be focused on this area. Otherwise, the medium complexity of the mobility could be due to the change in the functional mobility, for a lack of motor skill, and the protocols that would be applied would be in this area, different from the previous one.

The re-categorization process will depend on where the patient is hospitalized and on the agreed protocols in each unit. For example, if the patient is in the ICU, they are categorized every day, once a day, or when the patient’s condition changes. An example of this is in the cases of patients categorized in the morning and then a spontaneous ventilation test is performed, and when extubated, their ventilatory support condition changes, which again requires that they be re-categorized. In the case of patients undergoing neurorehabilitation, they are categorized once a week (every Wednesday) by applying a battery of evaluation scales that reveal their progress and determine the therapeutic load.

As an example, the matrix for categorizing Motor Rehabilitation among ICU patients is shown in Table 2 [11, 12], and the matrix for categorizing Adult Pulmonary Rehabilitation for patients with Invasive Mechanical Ventilation is shown in Table 3. These matrices were selected from among the various other matrices developed. 96,400 instances of inpatient care where the physician recommended physical therapy were categorized. This was from a total of 145,968 instances of inpatient care that were recorded throughout the duration of the study (17 quarters). These records were stored in a database in order to be analyzed on a quarterly basis. The use of this data was approved by the Scientific Ethics Committee of the Faculty of Medicine, Clinica Alemana of Santiago-Universidad del Desarrollo, who authorized the exemption requiring participants’ consent as the data was anonymized.

**Table 2:** Matrix for Categorizing Motor Rehabilitation with ICU patients

Variables	1 point	2 points	3 points
MRC-Sum Score^(a)^	60 to 48	47 to 37	36 to 0 / Not measurable
FSS-ICU ^(b)^	35 to 29	28 to 16	15 a 0 / Not measurable
Handgrip^(c)^	> 30kg (M)	27-30 kg (M)	< 27 kg (M)
	>18 kg (F)	15-18 kg (F)	< 15kg (F)
S5Q^(d)^	4 – 5	3	< 2
CATEGORIZATION			
Low Complexity		4 points	
Medium Complexity		5 – 8 points	
High Complexity		9 –12 points	

^**(a)**^ MRC-Sum Score = Scale for measuring muscle strength ^(b)^FSS-ICU = Scale for measuring functional status ^(c)^Handgrip = Measure of grip strength ^(d)^S5Q= Scale for assessing cooperation

**Table 3:** Matrix for Categorizing Adult Pulmonary Rehabilitation for patients with Invasive Mechanical Ventilation

Variables	1 point	2 points	3 points
Auscultation	Normal	Diminished lung sounds and/or wheezing, diffuse crepitation pulmonary edema	Abolished lung sounds and/or wheezing both phases and/or pulmonary edema bronchial breathing
Ventilatory assistance	Spontaneous	Assist-Control	Control
Amount of secretion	+	++	+++
Minute volume	< 7 L/Min	7 - 12 L/Min	> 12 L/Min
Peep^(a)^ / FiO2^(b)^	< 6 / 30	7-9 / 31-50	> 10 / 51
Static Compliance	Not measurable or > 50 cmH2O	49 - 35 cmH2O	< 34 cmH2O
CATEGORIZATION			
Low Complexity		6 points	
Medium Complexity		7 -12 points	
High Complexity		13-18 points	

^(a)^ Peep= Positive end expiratory pressure ^(b)^ FiO2= Fraction of inspired oxygen

**Fig. 1:**
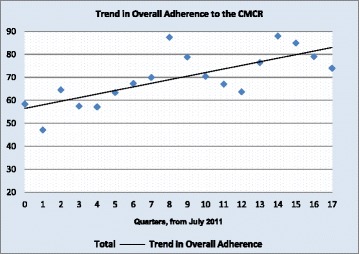
Trend in Overall Adherence to the CMCR. Key: (included in the graph)**.** The straight line depicts the trend in the data

The CMCR was rolled out via the hospital’s electronic patient records (EPR), in March 2011 through a coordinated effort in order to standardize the use of the model in clinical practice and optimize the data collection process. The results were then stored in the inpatient database and analyzed quarterly. Adherence to the CMCR was also used as a quality indicator as part of the hospital’s accreditation process (Fig. 1).

Meetings were held every semester with each of the corresponding teams to show their adherence level and design strategies for improvement. Strategies refer to re-evaluating if the chosen clinical variables were the correct ones for the clinical situation of the patients. The way in which the data were recorded was also evaluated and variations were made according to feedback from the teams, and finally in the quarterly team meetings, the need for retraining in the application of a specific scale that made use of the full categorization model was determined. Additionally, individual interviews were conducted to analyze individual-level variables that may have influenced adherence by healthcare professionals.

Throughout this time, the different matrices were put to the test by the rehabilitation teams and any necessary adjustments were made. The percentage use of the CMCR with ICU and non-ICU inpatients was recorded quarterly. The trend in these percentages between July 2011 and December 2015 was then analyzed.

### Statistical analysis

Trends in adherence were analyzed using a Prais-Winsten regression (a first-order autoregressive model) [13, 14]. The trends were compared using a repeated measures ANOVA for mixed models [15, 16], with a significance level of 0.05. The data was processed using STATA 13.0.

## Results

First, Fig. 1 shows the overall level of adherence per quarter (between July 2011 and December 2015). Adherence increased each quarter by an average of 1.48% (*p* = 0.005). Autocorrelation was around 30%, i.e., adherence in a given quarter had a 30% impact on the following quarter.

Second, Fig. 1 shows the results for the units with the highest and lowest levels of adherence to the model (ICU and non-ICU, respectively). Initial adherence with ICU patients was 63%, and increased by an average of 1.28% per quarter (*p* = 0.003). Meanwhile, initial adherence with non-ICU inpatients was 54%, with a quarterly increase of 1.62% (*p* = 0.007).

## Discussion

The results of this study show that physical therapists adhere to the use of standardized clinical assessments by means of a CMCR. Therapists working in the ICU showed earlier adherence than those not working in the ICU; however, the increase in non-ICU units was greater over time.

When interpreting the results, we noted that adherence sustained over time may be due to physical therapists being able to comparatively assess the advantage of having a model that standardized, arranged and promoted objective decision-making through the use of systematic and agreed assessment scales. The fact that the categorizations contain specific clinical variables carefully chosen by the teams is meant to reduce variability in clinical practice and ensure that the professionals observe the same, most important determinants affecting patients’ clinical behavior without their subjective criteria having a negative influence. In addition, we incorporated a cross-sectional nomenclature (high, medium and low complexity), which made the clinical evolution of a patient from high to low complexity easier to observe, and gave a therapeutic load (number of sessions) associated with the patient’s functional needs, showing intervention-related clinical improvement. Another implication of the CMCR is related to the allocation of human resources; when the demand for services increases, we prioritize care for the patients categorized as high and medium complexity over the patients of low complexity, since the clinical variables incorporated into this complexity are less severe, which makes it possible for us to optimize the human resources and redistribute them without putting the patients at risk. Finally, its simple design made its daily use easier.

The characteristics mentioned for the CMCR overcame the barriers described by the literature for the use of assessment scales by physical therapists in clinical practice [17].

The data analysis was performed using a database that contains all the care services provided by physical therapists over 17 quarters. Adherence was assessed quarterly, and the percentage of the instances of inpatient care categorized from the total number of instances of inpatient care performed per quarter was obtained. There is no individual patient follow-up; the result shows the overall adherence of all the care provided over a period of time.

**Fig. 2:**
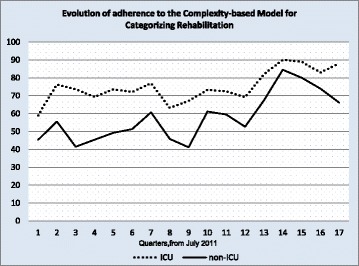
Adherence with adult ICU patients and non-ICU inpatients. Key: (included in the graph). The dashed lined shows adherence with ICU patients. The solid line shows adherence with non-ICU inpatients

Sustained adherence to the study during the 17-month follow-up was also relevant. Most studies about sustainability published in the literature show its decrease after a year of any kind of implementation [18]. We believe the successful sustainability shown in this study is due to two important strategies: first, auditing and feedback to professionals, and second, linking the CMCR as a quality indicator in the national and international accreditation processes in which our institution participates. This was meant to align the implementation strategies with the hospital’s organizational goals, a concept referred to by Scheck McAlearney as “organizational coherence” [19]. This is clearly visible when analyzing the peaks in adherence over the 5-year period of the study as they coincide with the accreditation processes conducted within the organization (Fig. 2).

When analyzing adherence with ICU and non-ICU inpatients separately, we noted a higher level of initial adherence with ICU patients. This behavior is in line with other studies that have suggested that patients managed in closed intensive care units by well-trained staff enjoy more positive results that non-ICU inpatients [20]. Therefore, the ICU would appear to be a favorable environment for implementing new clinical tools.

The implication here is that there is likely a greater concentration of innovators in intensive care units who bring new ideas to the system and who, in turn, become leaders in disseminating innovation [21–24].

The goal in recent years has been to incorporate the most up-to-date clinical care, integral, interdisciplinary and holistic vision required for this kind of intervention effectively. Locally in Chile, the challenge is much greater: not only do we have to install the rehabilitation model in regular clinical care, but we also have to set it up from the start of the acute health care intervention (ICU, intermediate), and we must generate models capable of standardizing evaluations, interventions, protocols, outcomes, goals and follow-up. Despite the difficulties this can imply, the movement towards innovation is more likely to become a reality if the vision is shared and consistent with the institutional culture and organization policy. From this point of view, the CMCR can be a concrete alternative for achieving this goal.

The main limitation of this study is the lack of evidence regarding the implementation of clinical models involving standardized assessments in rehabilitation. Another limitation of the work is that other rehabilitation groups were not analyzed because the greatest percentage of professionals (90%) are physical therapists, and it was decided that the implementation should begin with the most representative rehabilitation group. Thus, no study was made of the individual variables that influence the CMCR in this group of professionals, but rather their use through the clinical records. Our article focuses mainly on the behavior of the physical therapists with respect to the use of the CMCR as a clinical tool, since for successful implementation and to see its impact on patients, we needed to ensure that the greatest percentage of professionals was using it. We hope in future investigations to be able to show the collected data of the impact on the patients.

## Conclusion

The CMCR facilitated adherence to standardized assessments by physical therapists in an acute hospital. Furthermore, the strategies adopted during the implementation of the model and for its long-term sustainability were successful throughout the duration of this study (17 quarters). Adherence continuously increased over time with both ICU and non-ICU inpatients, with the increase for ICU patients being slightly greater.

The results suggest that intensive care units provide a favorable environment for the use of standardized assessment scales. Linking an innovation to the organization’s internal processes, such as Quality Accreditation, should also be considered as a fundamental element when designing such a tool.

## Additional file

Additional file 1: Database CMCR. (XLSX 5317 kb)

## Abbreviations

APTA: American Physical Therapy Association
CMCR: Complexity-based Model for Categorizing Rehabilitation
EPR: Electronic patient records
ICU: Intensive care united
